# A Case of Abdominal Aortic Retroperitoneal and Mesenteric Amyloid Light Chain Amyloidoma

**DOI:** 10.1155/2016/4146030

**Published:** 2016-09-26

**Authors:** Kazuhiro Yokota, Dai Kishida, Hidekazu Kayano, Masahide Yazaki, Yuki Shimada, Yuji Akiyama, Toshihide Mimura

**Affiliations:** ^1^Department of Rheumatology and Applied Immunology, Faculty of Medicine, Saitama Medical University, Saitama, Japan; ^2^Department of Internal Medicine (Neurology and Rheumatology), Shinshu University School of Medicine, Nagano, Japan; ^3^Department of Pathology, Saitama Medical University, Saitama, Japan; ^4^Ogawa Red Cross Hospital, Saitama, Japan

## Abstract

We report the case of a Japanese woman with amyloid light chain (AL) amyloidoma in the abdominal aortic retroperitoneum and mesentery. Irregular soft tissue mass lesions with calcification in the abdominal aortic retroperitoneum and mesentery were initially detected by computed tomography at another hospital. The lesions gradually compressed the duodenum, causing symptoms of bowel obstruction. The patient was clinically diagnosed with retroperitoneal fibrosis, and prednisolone was administered at a dose of 40 mg/day. However, the lesions did not change in size and her symptoms continued. She was transferred to our hospital and underwent mesenteric biopsy for histopathology using abdominal laparotomy. The histopathological and immunohistological findings of the mesenteric specimen demonstrated lambda light chain deposition. Accordingly, the patient was finally diagnosed with AL amyloidoma with no evidence of systemic amyloidosis. After laparotomy, her general condition worsened because of complications of pneumonia and deep vein thrombosis. She died suddenly from acute myocardial infarction. We have concluded that abdominal aortic retroperitoneal and mesenteric AL amyloidoma may have very poor prognoses in accordance with previous reports. In addition, the size and location of AL amyloidoma directly influence the prognosis. We suggest that early histopathology is important for improving prognosis.

## 1. Introduction

Amyloidosis is a disease that occurs as a consequence of abnormal insoluble protein (amyloid) deposits in the organs and/or tissues [1]. Amyloidosis is classified into two types based on the site of amyloid deposition. One type is systemic amyloidosis, which is characterized by amyloid deposits in the systemic organs and/or tissues, while the other type is localized amyloidosis, which is characterized by amyloid deposits in a specific organ or tissue. In addition, amyloidosis can be classified according to the amyloid precursor proteins involved [2]. A large proportion of these proteins are amyloid light chain (AL) and amyloid A (AA). AL amyloidosis is characterized by the deposition of amyloid fibers that consist of monoclonal immunoglobulin (M protein) light chains, which are produced by abnormal plasma cells. Cases unaccompanied by plasma cell neoplasms (solitary plasmacytoma or multiple myeloma) and primary macroglobulinemia are known as primary AL amyloidosis. In most cases of AL amyloidosis, serum M protein, urine Bence Jones protein, or serum free light chain are detected [3]. The median survival time of patients with AL amyloidosis is 2 years [4].

Reactive AA amyloidosis is characterized by deposition, predominantly in the gastrointestinal tract and kidneys, of metabolite amyloid A originating from serum amyloid A. Serum amyloid A is produced by the liver and promoted by interleukin- (IL-) 6 in response to chronic inflammatory disorders like rheumatoid arthritis, tuberculosis, osteomyelitis, and so on. Therefore, inflammatory markers like serum C-reactive protein and serum amyloid A levels respond with a high sustained value. In the past, the 5-year survival rate was 50% [5], but, with the increased use of anti-IL-6 receptor antibody (e.g., tocilizumab), improvements in survival are expected [6].

Amyloidoma (tumoral amyloidosis) is a solitary localized tumor-like amyloid deposition defined as the absence of any evidence of systemic amyloidosis. In many cases, the development sites of amyloidoma are in the systemic organs such as the respiratory system [7], genitals [8], gastrointestinal tract [9], and central nervous system [10]. However, development in the soft tissues, the retroperitoneum and mesentery in particular, is quite rare [11–13]. Here, we report a case with a literature review in order to characterize the disorder and report on its treatment and prognosis.

## 2. Case Report

**Figure 1:**
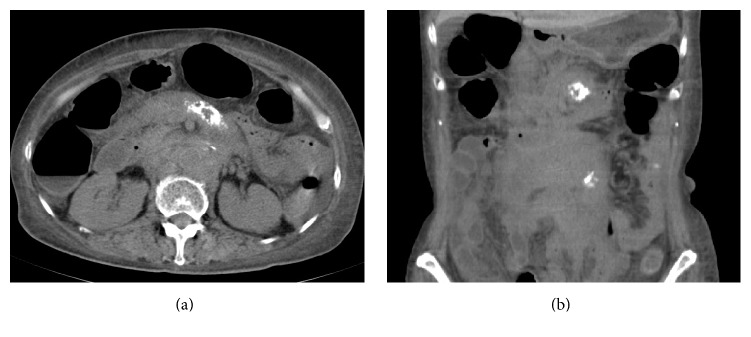
Abdominal computed tomography showing irregular soft tissue tumor masses with calcification in the abdominal aortic retroperitoneum and mesentery.

A 65-year-old Japanese woman was admitted to our hospital with a 1-month history of vomiting. She had undergone no medical examination for 10 years prior to admission. Nine months before admission, she was hospitalized at another hospital with dyspnea. She was diagnosed with acute heart failure and three-vessel coronary arteriosclerotic lesions by coronary angiography. She subsequently underwent coronary artery bypass grafting surgery. At that time, computed tomography (CT) revealed irregular soft tissue mass lesions with calcification in the abdominal aortic retroperitoneum and mesentery (Figures 1(a) and 1(b)). Positron emission tomography (PET) showed that ^18^F-fluorodeoxy glucose mildly accumulated only in the irregular soft tissue mass lesions (standardized uptake value 3.35). Based on the PET result, the soft tissue mass lesions were considered unlikely to be cancerous, and she was followed up by regular imaging evaluation.

One month before admission to our hospital, the patient was readmitted to the previous hospital with persistent vomiting. Abdominal CT revealed enlargement of the abdominal aortic retroperitoneal and mesenteric soft tissue tumor masses. The masses were compressing her duodenum, leading to symptoms of bowel obstruction. She was clinically diagnosed with retroperitoneal fibrosis, and prednisolone was administered at a dose of 40 mg/day. After 3 weeks of treatment, however, the tumor masses did not reduce in size and her symptoms persisted.

She was transferred to our hospital at this point, and on admission she was afebrile (35.3°C), her body weight was 45.1 kg (no weight loss), and she had a performance status of 3. The palpebral conjunctiva appeared anemic, although we observed no marked hair loss, oral ulcers, tongue swelling, thyroid gland and lymph node enlargement, skin rash, arthritis, or Raynaud's phenomenon. The soft tissue masses were not palpable on the abdomen. In addition, there were no findings of syncope, dizziness on standing up, movement disorders, or sensory impairment. The results of laboratory examinations were as follows: white blood cell count increased to 14,750/*μ*L, hemoglobin level decreased to 9.5 g/dL, platelet count was normal 302,000/*μ*L, and erythrocyte sedimentation rate elevated to 21 mm/h. Her serum total protein and albumin concentrations decreased to 5.4 g/dL and 3.1 g/dL, respectively. Her serum protein fraction revealed that her albumin level was decreased to 57.0%, *α*1-globulin increased to 5.0%, and *α*2-globulin (13.6%), *β*-globulin (8.7%), and *γ*-globulin (15.7%) were all normal. Her serum IgG (1,512 mg/dL) and IgG4 (10 mg/dL) were normal. Her serum IgA decreased to 68 mg/dL. Her serum concentrations of IgM (70 mg/dL), C-reactive protein (0.1 mg/dL), serum creatinine (81.3 *μ*mol/L), calcium (2.32 mmol/L), glycosylated hemoglobin (5.7%), and total cholesterol (188 mg/dL) were normal. She was positive for antinuclear antibody with a titer of 1 : 40, and her serum soluble interleukin-2 receptor level was increased to 662 U/mL. Urinalysis was normal with no Bence Jones protein. Electrocardiography revealed no abnormalities including axis deviation, bundle branch block, or low voltage. In addition, there was no granular brightness in the ventricular wall on cardiac ultrasound. No osteolytic lesion and compression fracture of the skull, femoral neck, or thoracolumbar spine were observed by whole-body CT.

**Figure 2:**
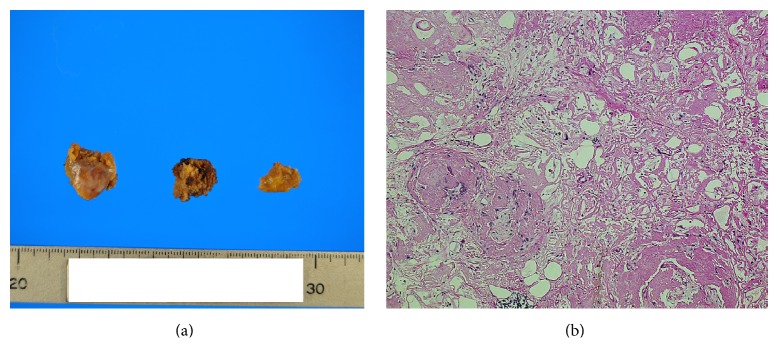
Biopsy of the mesenteric lesion showing poor cellular components and hyalinized, degenerated tissue with a small amount of fat. No evidence of the infiltration of inflammatory cells and tumor cells ((a) macrograph, (b) hematoxylin and eosin stain: objective lens, ×20).

After being transferred to our hospital, the patient continued to take prednisolone at a dose of 40 mg/day. However, the masses still did not reduce in their size and her symptoms were still persistent. Therefore, on the 40th day of hospitalization, we performed mesenteric biopsy for histopathology and decided to attempt gastrointestinal bypass surgery using abdominal laparotomy. The intraoperative findings showed that the mesentery at the root of the superior mesenteric artery was very hard and thick with white coloration and was compressing the third portion of the duodenum. In addition, two fibrotic masses approximately 1 cm in diameter were found in the mesenteric lesion (Figure 2(a)). The fibrotic mass was resected and dissected to yield a histopathological and immunohistological specimen. Unfortunately, gastrointestinal bypass surgery could not be performed because her hemodynamics were unstable during the operation. At that time, postoperative finding was mesenteric panniculitis.

**Figure 3:**
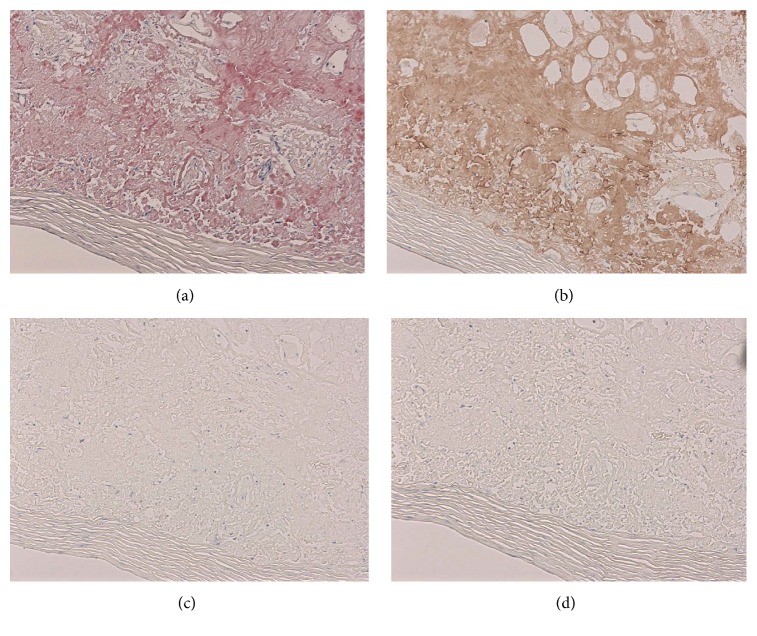
Congo red staining demonstrating amyloid deposition ((a) objective lens, ×10). Immunohistological staining revealing positivity for lambda light chain ((b) objective lens, ×10) and negativity for kappa or transthyretin ((c), (d) objective lens, ×10).

The result of a biopsy of the resected mesenteric lesion indicated poor cellular components and hyalinization and degeneration of tissue with a small amount of fat. No evidence of the infiltration of inflammatory or tumor cells was present (Figure 2(b)). Congo red staining demonstrated amyloid deposition (Figure 3(a)). In addition, immunohistological staining [14] was positive for lambda light chains (Figure 3(b)) but negative for kappa or transthyretin (Figures 3(c) and 3(d)). In addition, electrophoresis of serum and urine proteins showed a monoclonal spike in the gamma region, which indicated IgG and Bence Jones protein lambda light chains. A gastric mucosal biopsy and random skin biopsy showed no evidence of amyloid deposition. There were no symptoms or physical, laboratory, or imaging findings of systemic amyloidosis. Accordingly, we finally diagnosed the patient with AL lambda amyloidoma in the abdominal aortic retroperitoneum and mesentery. After laparotomy, her general condition worsened due to complications of pneumonia and deep vein thrombosis. We scheduled bone marrow aspiration as soon as possible, but she experienced sudden acute myocardial infarction and died 5 months after admission.

## 3. Discussion

In the present case, soft tissue masses occurred in the abdominal aortic retroperitoneum and mesentery and proved difficult to diagnose. We finally diagnosed AL lambda amyloidoma based on a mesenteric biopsy using abdominal laparotomy.

Amyloidoma developing in soft tissues, the retroperitoneal and mesentery in particular, is quite rare. To the best of our knowledge, eight cases of retroperitoneal and mesenteric AL amyloidoma have been reported [11–13], including the present case (Table 1). These reports showed that the mean patient age was 66 years (range, 48–79 years). Four of eight patients were men. The tumor diameter of seven of eight patients was more than 10 cm; however, data on one patient were not available. In addition, three of eight patients had lymphoproliferative disorders, two had multiple myeloma, and one had plasmacytoma; however, data on the other patient were not available. In terms of treatment, four out of eight patients received chemotherapy regimens which were not described (combined with surgical resection in one case), two received glucocorticoid monotherapy, and one received radiation therapy; again, data on one patient were not available.

In the five patients who underwent chemotherapy or radiation therapy, partial remission was achieved in a short period. In one case, death occurred shortly after glucocorticoid monotherapy. These results show that retroperitoneal and mesenteric amyloidoma are partially affected by chemotherapy or radiation therapy but not glucocorticoid monotherapy. Our patient unexpectedly died of the incidence of acute myocardial infarction. However, she would have been able to live a little longer if she had been able to receive intensification therapy such as chemotherapy for amyloidoma. It is noteworthy that these reports demonstrated a median survival time of 11.8 months, with no evidence of survival for more than 2 years. Thus, all previous reports suggest that retroperitoneal and mesenteric amyloidoma are associated with extremely poor prognoses.

A few reports have shown that AL amyloidoma did not recur for several years after surgical complete resection [15, 16]. However, AL amyloidoma is difficult to diagnose in the earlier stage because of the absence of specific subjective symptoms in most cases [17]. Consequently, the disease is often found to have progressed to an irreversible stage when amyloidoma was found. Shiels et al. reported a case of pelvic AL amyloidoma with a diameter of more than 20 cm that could not be treated by surgical resection [18]. The patient died before the start of chemotherapy or radiation therapy. Similarly, in our case, the patient was incidentally diagnosed with retroperitoneal and mesenteric AL amyloidoma with a diameter of more than 15 cm. As in the previous case, she could not undergo surgical resection. Therefore, we suggest that the size and location of the AL amyloidoma directly influence prognoses. Complete surgical resection before tumor size increases and earlier chemotherapy or radiation therapy are important for improving the outcomes of patients with retroperitoneal and mesenteric AL amyloidoma.

In the present case, we suspected that plasmacytoma was the cause of AL amyloidoma because the electrophoresis of serum and urine proteins revealed the presence of M protein, which was indicative of IgG and Bence Jones protein lambda light chains. We also considered multiple myeloma as a potential underlying cause. However, the following points contradicted this hypothesis: (1) the patient's total serum protein level was slightly decreased; (2) her M protein level was only slightly increased; (3) although serum IgA was slightly below the normal range, it was less likely that our case had multiple myeloma because the serum level of IgM was within normal limits; (4) there was no evidence of organ damage such as osteolytic lesions and renal insufficiency, which were present in previous case reports of multiple myeloma associated with amyloidoma [11, 13]; and (5) PET revealed that ^18^F-fluorodeoxy glucose mildly accumulated only in the abdominal aortic retroperitoneal and mesenteric lesions. Therefore, as there was no evidence of plasmacytoma in the biopsy of the resected mesenteric lesion, it was possible that the plasmacytoma in the intraperitoneal soft tissue lesions proliferated in the neoplastic stage and produced M protein locally. This M protein was then deposited in the abdominal aortic retroperitoneum and mesentery, causing the irregular soft tissue masses.

In conclusion, this is a first report of retroperitoneal and mesenteric AL amyloidoma, a very rare condition that shows poor prognosis. Of special importance is that the size and location of AL amyloidoma directly influence the prognosis. We suggest that early histopathology is essential for improving prognosis. In addition, once AL amyloidoma is diagnosed, complete surgical resection, chemotherapy, or radiation therapy should be considered promptly.

## Figures and Tables

**Table 1:** Case reports of abdominal aortic retroperitoneal and mesenteric amyloid light chain amyloidoma.

Age · sex	Mass: location	Tumor diameter	Amyloid type	Diagnosis	Therapy	Outcome (months from presentation)	Reference
63 · male	Mesentery	>13	AL(*λ*)	Lymphoproliferative disorder	Surgery (partial excision) chemotherapy	Dead (17)	[11]
48 · male	Mesentery	10	AL(*λ*)	—^**∗**^	—^**∗**^	—^**∗**^	[11]
66 · female	Mesentery	>15	AL(*λ*)	Lymphoproliferative disorder	Chemotherapy	Dead (13)	[11]
73 · male	Mesentery mediastinum	>10	AL(*λ*)	Lymphoproliferative disorder	Chemotherapy	Dead (5)	[11]
68 · female	Retroperitoneum	—^**∗**^	AL(*λ*)	Multiple myeloma	Chemotherapy	Dead (8)	[11]
67 · male	Retroperitoneum	10	AL(*κ*)	Plasmacytoma	Radiation therapy	—^**∗**^	[12]
79 · female	Mesentery	>10	AL(*κ*)	Multiple myeloma	Steroid	Dead (14)	[13]
65 · female	Mesentery retroperitoneum	15	AL(*λ*)	Plasmacytoma suspected	Steroid	Dead (14)	Our case

*∗* indicates not described.
